# Supplementary figures and images for: The BICD2 dynein cargo adaptor binds to the HPV16 L2 capsid protein and promotes HPV infection
Source: PLoS Pathog. 2024 Jun 3;20(6):e1012289. doi: 10.1371/journal.ppat.1012289 (PMC11230635; doi:10.1371/journal.ppat.1012289)

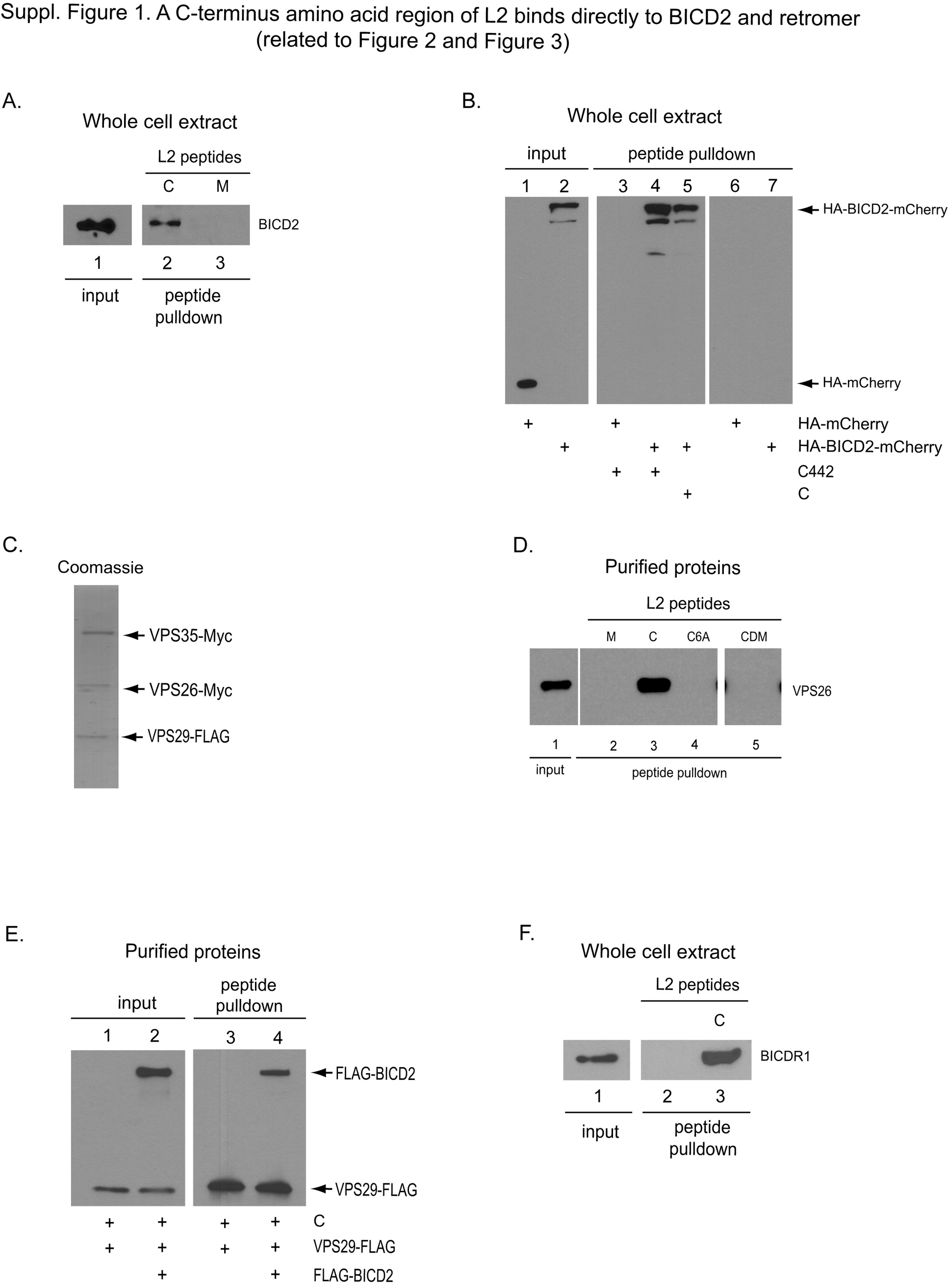

Supplement: S1 Fig — A. Whole cell extracts derived from HeLa cells were incubated with biotinylated L2 peptide C or M. The peptides were precipitated by streptavidin beads and the precipitated material was subjected to SDS-PAGE and immunoblotting with an antibody recognizing BICD2 to detect endogenous BICD2. B. As (A) except using whole cell extracts derived from HeLa cells transfected with HA-BICD2-mCherry or the control HA-mCherry with biotinylated L2 peptide C or C442, or no peptide as a control. C. Coomassie stain of the retromer complex (VPS29-FLAG, VPS35-Myc, and VPS26-Myc). D. As (A) except using purified retromer with the indicated biotinylated L2 peptide (please see peptides in Fig 2C). The precipitated material was subjected to SDS-PAGE and immunoblotted with an antibody recognizing VPS26. All samples were electrophoresed on the same gel. Irrelevant lanes were removed. E. Biotinylated peptide C was incubated with the retromer complex in the presence or absence of FLAG-BICD2. The peptide was precipitated as described above and immunoblotting with an antibody recognizing FLAG was performed. VPS29-FLAG indicates the presence of the retromer complex in the pellet. F. As in (A) except using only biotinylated L2 peptide C or no peptide as a control and immunoblotting for endogenous BICDR1. (TIF) [file ppat.1012289.s001.tif]

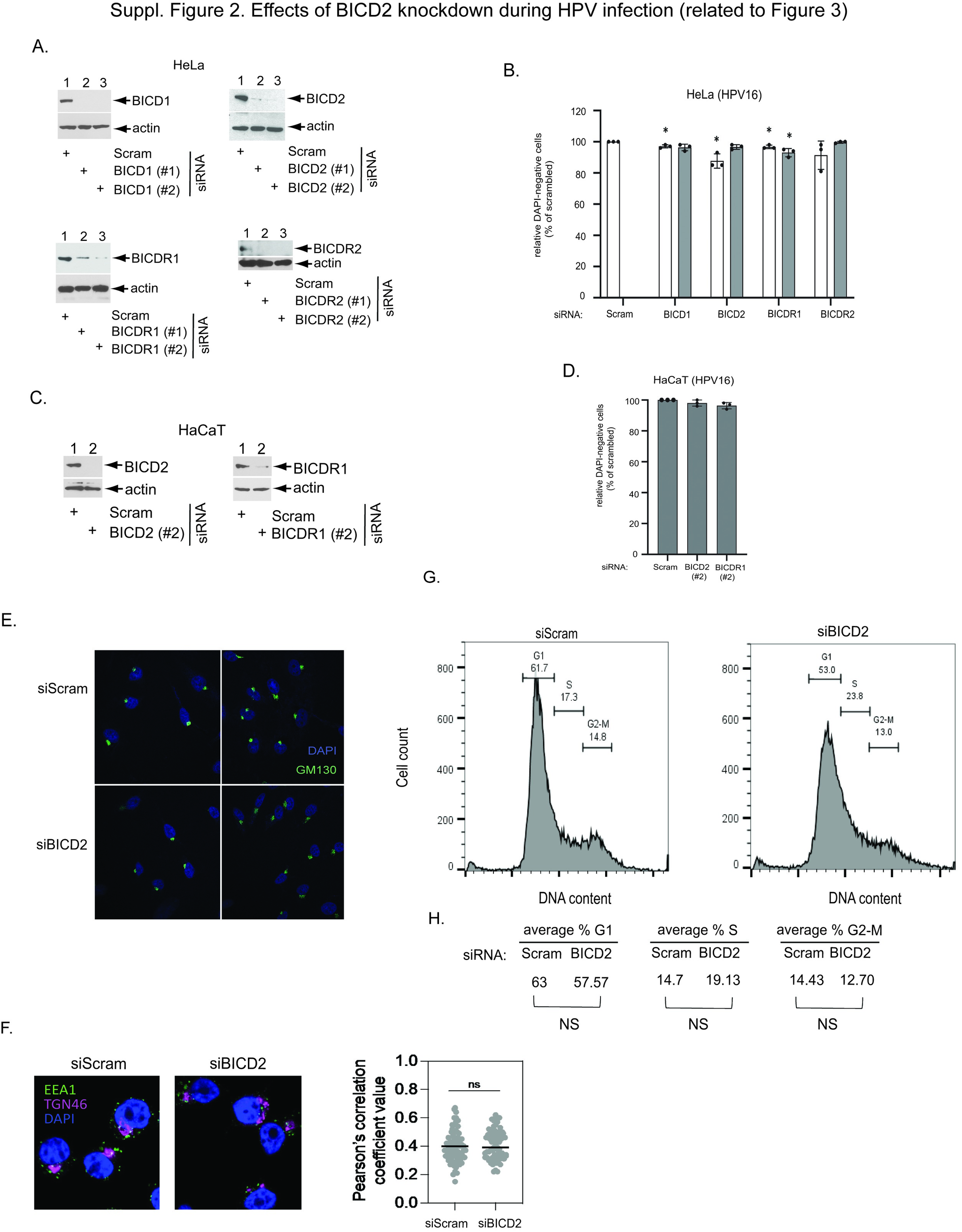

Supplement: S2 Fig — A. Whole cell extracts derived from HeLa cells transfected with indicated siRNA were subjected to SDS-PAGE and immunoblotting with the indicated antibodies. Actin, loading control. B. HeLa cells transfected with the indicated siRNA were trypsinized and incubated in a buffer containing DAPI. The fraction of DAPI-negative cells was measured by flow cytometry. The results were normalized to the fraction of untreated, DAPI-negative cells. *p ≤ 0.05 C. As in (A), except using HaCaT cells. D. As described in (B), except using HaCaT cells. E. HeLa cells transfected with the indicated siRNA were fixed, permeabilized, and subjected to immunofluorescent staining with an antibody recognizing GM130 (green). Nuclei were stained with DAPI (blue). Representative images taken by confocal microscopy are shown. F. HeLa S3 cells were transfected with scrambled control (siScram) or BICD2-targeting (siBICD2) siRNAs. Cells were stained with antibodies recognizing EEA1 and TGN46. Immunofluorescence images were shown; EEA1, green; TGN46, magenta; nuclei (DAPI), blue. Pearson’s correlation coefficient values for EEA1 and TGN46 colocalization in those cells are shown. Each dot represents an individual cell (n>30) and black horizontal lines indicate the mean value of the analyzed population in each group. ns, not. significant. The graph shows results of a representative experiment. Similar results were obtained in two independent experiments. G. HeLa cells transfected with the indicated siRNA were stained for cellular DNA by incubation with Hoechst 33342 then trypsinized and analyzed by flow cytometry for relative Hoechst 33342 fluorescence. One set of representative histograms is shown from three independent experiments, with the percentage of cells in G1, S, or G2-M phases indicated. H. Means of cell cycle phase distributions as in (F) from three independent experiments. N.S., not significant. BICD2 siRNA #2 was used in panels E-F. (TIF) [file ppat.1012289.s002.tif]

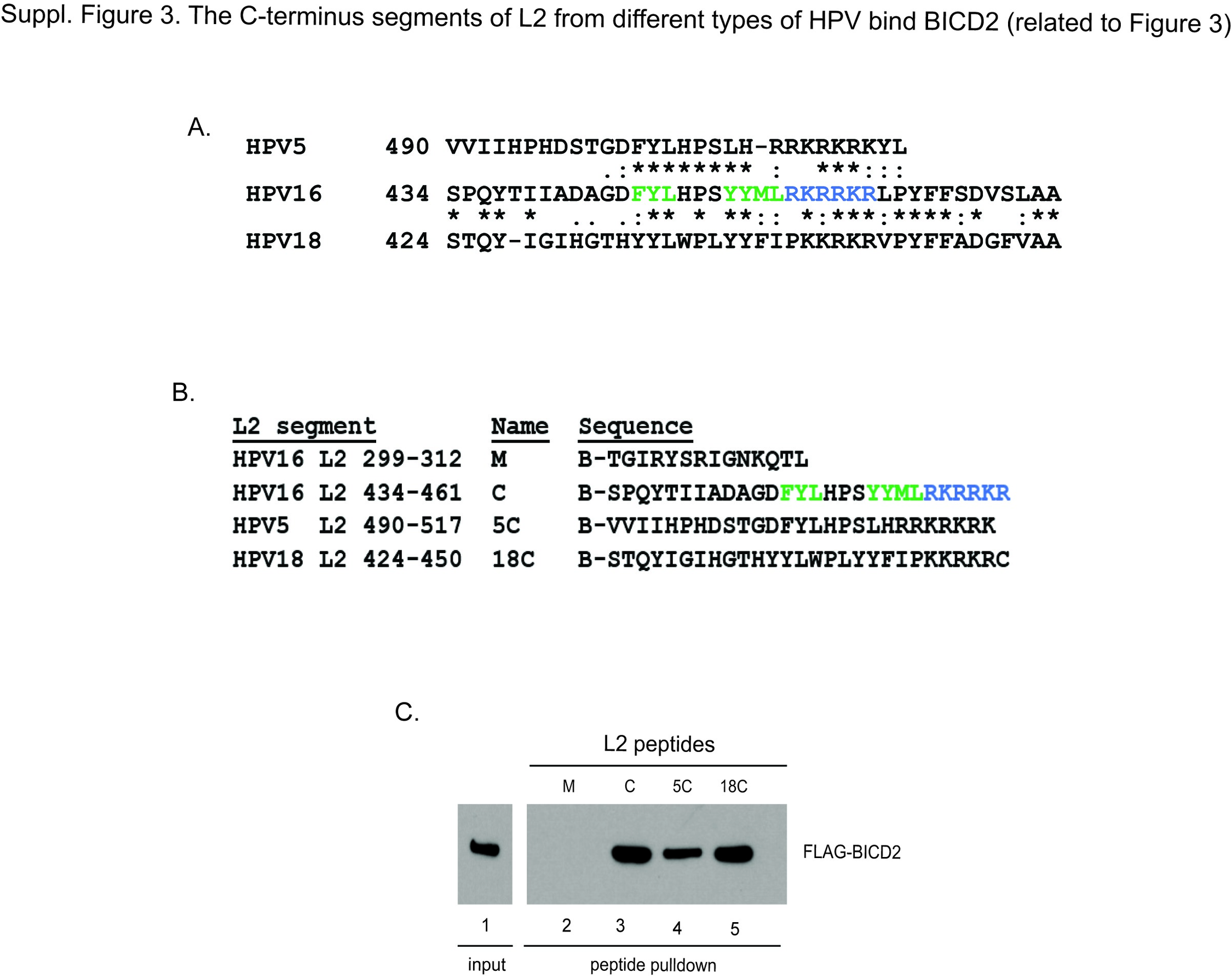

Supplement: S3 Fig — A. L2 amino acids of HPV5 and HPV18 were aligned with HPV16 L2 C-terminus segment 434–473. The asterisk indicates identity, colon indicates conservative substitution, and period indicates semi-conservative substitution. B. Sequences of the L2 peptides of HPV16, HPV5 and HPV18. B indicates biotin. In HPV16 peptide C, amino acids for the retromer binding site are shown in green and those for the CPP are shown in blue. C. FLAG-BICD2 was incubated with the indicated biotinylated L2 peptides. Precipitation was performed using streptavidin beads. The precipitated material was subjected to SDS-PAGE and immunoblotted with an antibody recognizing FLAG. (TIF) [file ppat.1012289.s003.tif]

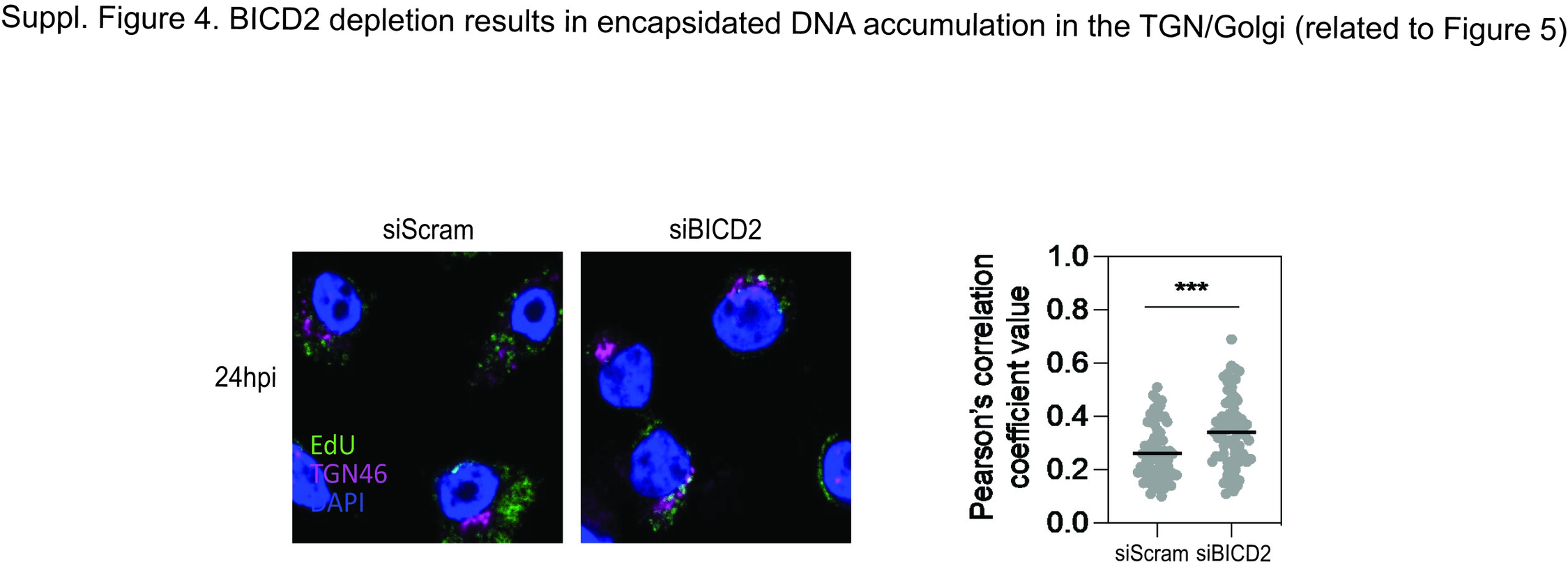

Supplement: S4 Fig — HeLa S3 cells were transfected with siScram or siBICD2 siRNA and infected with EdU-labeled HPV at the MOI of ~100. At 24 hpi, cells were stained with antibodies recognizing TGN46, and EdU was detected as described in Materials and Methods section. Immunofluorescence images were shown; EdU, green; TGN46, magenta; nuclei (DAPI), blue. Pearson’s correlation coefficient values for EdU and TGN46 colocalization in those cells are shown. Each dot represents an individual cell (n>30) and black horizontal lines indicate the mean value of the analyzed population in each group. ***p < 0.001. The graph shows results of a representative experiment. Similar results were obtained in two independent experiments. BICD2 siRNA #2 was used. (TIF) [file ppat.1012289.s004.tif]
