# Supplementary material for: The BICD2 dynein cargo adaptor binds to the HPV16 L2 capsid protein and promotes HPV infection
Source: PLoS Pathog. 2024 Jun 3;20(6):e1012289. doi: 10.1371/journal.ppat.1012289 (PMC11230635; doi:10.1371/journal.ppat.1012289)
Supplement: Attachment — Submitted filename: Speckhart et al. (Point-by-point response).docx [file ppat.1012289.s005.docx]

**Major Issues: Key Experiments Required for Acceptance**

**Reviewer #1**:

Major Points:

1. The peptide binding data convincingly shows that BICD2 can bind to a 20-mer peptide from L2. A missed opportunity was to further dissect this binding and define the specific residues of L2 that necessary, with the hope of identifying a mutant that is specifically defective for binding to BICD2 but otherwise functional for retromer binding and membrane spanning. The conclusion that this small region is sufficient for dynein/BICD2 binding would be solidified if the in vitro binding assays from Figure 2 were repeated with mutation of the larger recombinant L2 (delta1-67) fragment, and with mutant virions during infection. Do peptides #3 and #4 still bind BICD2 if the native C-terminal sequences are present? Can these peptides simultaneously bind retromer and BICD2/dynein?

Response: These are important questions. To dissect the C-terminal segment, we performed new peptide pulldown experiments using L2 peptides with mutations in either the retromer binding site or the sequence encoding the cell penetrating peptide (CPP). We found that both the retromer binding site and the CPP are important for BICD2 binding (**new Figure 2E**) and for retromer binding (**new Supplemental Figure 1D**). These mutations have previously been tested in the context of mutant virions during infection (PMID: 25693203, PMID: 30122350). However, because retromer binding and CPP activity is required for stable insertion of L2 into the cytoplasm (PMID 34193420), we cannot yet establish if the inability of the mutant virions to infect is due to the lack of BICD2 binding as these mutations disrupt steps upstream of BICD2 binding. We do note, however, that binding of BICD2 to this segment is not mediated by binding of this segment to retromer, because purified BICD2 binds.

Additionally, we tested L2 peptides that include the native C-terminal sequences downstream of the CPP as requested and showed that they are still able to bind BICD2 efficiently (**new Figure 2G**).

To address if these peptides simultaneously bind retromer and BICD2, we performed the peptide pulldown after incubating the peptide with a mixture of purified retromer and BICD2. We found that both retromer and BICD2 were pulled down (**new Supplemental Figure 1E**). This could indicate that this peptide simultaneously binds retromer and BICD2. Alternatively, some peptides in the reaction could bind retromer while others bind BICD2. Because a single viral particle may have multiple copies of L2 inserted across the endosomal membrane, each capsid could bind to BICD2 and retromer at the same time even if this L2 segment cannot bind both proteins simultaneously. In this scenario, some cytosolic exposed L2 molecules could bind retromer while other L2 molecules bind BICD2.

2. The conclusion that HPV trafficking is blocked at both the endosome and Golgi is confusing and only relies on PLA data. An important control that is missing is to determine if BICD2 silencing alters the distribution of either EEA1 or TGN46. With regards to TGN46, this is a transmembrane protein localized to Golgi at steady state but can be secreted to the plasma membrane and re-enter via endosomes before retrieval back to the Golgi, quite different than the GM130 Golgi matrix protein that doesn’t appear to change distribution upon BICD2 knockdown (Fig S2E). If BICD2 silencing causes more TGN46 protein to be within endosomes (increases TGN46 + EEA1 PLA and/or localizes TGN46 to endosomes by conventional IF microscopy) then that could greatly affect the interpretation of the PLA data. Alternate methods to look at trafficking may also strengthen the paper.

Response: As requested, we have now included immunofluorescence data showing that BICD2 knockdown does not affect EEA1 and TGN46 distribution or colocalization (**new Supplemental Figure 2F**). We agree with the importance of using an additional method to assess trafficking. We have now included data using PsV containing EdU-labeled DNA. Using this labeled virus, we found that the virus is trapped in the Golgi under BICD2 KD (**new Supplemental Figure 4**), consistent with the PLA data.

3. Ciliobrevin D treatment should perturb mitosis, which is needed for post-Golgi trafficking and infection. Proper cell cycle analysis and a growth curve in the presence of drug is needed to rule out this possibility. Another dynein inhibitor called dynarrestin was reported to induce spindle misorientation and pseudoprometaphase delay (PMID 29396292), so these drugs should be used cautiously, or limited to studies on early pre-mitotic trafficking. On the topic of cell cycle, the data in S2F show a difference in G2/M/G1 ratios upon BICD2 knockdown, although these did not reach statistical significance. The histograms shown in S2F have a different shape, and there is an additional shoulder of cells with >2n DNA content upon silencing of BICD2. I suggest a more rigorous analysis to ensure these cells are cycling properly through mitosis, with normal kinetics. Perhaps a cellular growth curve comparing siScram to siBICD2, and giving the siRNA more time to work before doing the cell cycle analysis. Dynein plays a role in mitosis and it seems that silencing an adaptor like BICD2 would perturb the kinetics of mitosis.

Response: We understand the concern regarding the use of ciliobrevin D. Because these results have been published by other labs (PMID: 33974675) and are not central to our conclusions, we have removed these figures from our manuscript. We have now included here further cell cycle experiments to more rigorously assess if BICD2 KD perturbs mitosis. We examined the cell cycle under similar conditions used in Figure 4B. Specifically, the knockdown was carried out for 24 hours at 10 nM siRNA, washed, and cell cycle was analyzed 48 hours later. There was no statistical difference between the control and knockdown cells (see histogram). These data suggest that the length of siRNA treatment does not alter the cell cycle progression. However, we agree that there is a slight shift in the histogram shape and thus cannot rule out a mild perturbation to mitosis. It is important to note that we observe defects in viral trafficking at 16 hpi that is prior to the time of nuclear entry, which is the mitosis-dependent step of HPV entry.

**Reviewer #2**:

Major Points:

1. The authors identified a 19-amino acid (442-461) C-terminal segment of HPV16 L2 that is sufficient for binding to BICD2. How conserved is the BICD2 binding region of L2 among HPV types? It would be helpful to show the alignment of this region among different HPV types. It would be interesting to know which 19 amino acid (s) are key binding epitopes for BICD2.

Response: Thank you for this suggestion. We have now added the alignment of this region in the revised manuscript (**new Supplemental Figure 3A**). We show that the corresponding segments of HPV18 and HPV5 L2 also bind BICD2 (**new Supplemental Figure 3C**). As noted in the response to the first point of reviewer #1, to address which amino acids are key BICD2 binding epitopes, we performed the peptide pulldown experiments using L2 peptides with mutations in either the retromer binding site or the cell penetrating peptide. We found that both the retromer binding site and the CPP are important for BICD2 binding (**new** **Figure 2E**). More detailed mutational analysis outside of the scope of this paper may allow dissection of these sites.

2. It would be interesting to know how HPV L2 without BICD2 binding epitopes infects and transports inside cells.

Response: We agree that this is a very interesting question. However, it is not possible to do this experiment with the mutants currently in hand because mutations that interfere with the retromer binding site or the CPP prevent exposure of L2 into the cytoplasm.

**Reviewer #3**:

Major Points:

1. A major concern is that the presented model remains for a good part speculative and unsubstantiated. The authors propose that BICD2/dynein already engage L2 upon membrane penetration in endosomes to facilitate transport to the TGN, and BICD2/dynein would promote budding of vesicles from the TGN at mitosis onset to facilitate nuclear import. While the first part is partially supported by the data showing that BICD2 knockdown reduces in part trafficking to the TGN, it remains unclear whether other adaptor proteins assist or compensate this transport step.

Response: We have now included data demonstrating that the HPV16 L2 peptide (named C) can interact with another dynein adaptor BICDR1 in cell lysate (**new Supplemental Figure 1F**). This result suggests that, in the case of HPV16 infection, adaptor proteins other than BICD2 may assist in trafficking steps. We discuss this result more fully in the manuscript. We have not identified adaptors other than BICD2 involved in entry of HPV5 and HPV18. It would be interesting to follow up on these results, although this is beyond the scope of this manuscript. We did not mean to imply that the BICD2/L2 interaction promotes budding of HPV during virus entry and have modified the text and figure to make that point clearer.

2. For the second part, the authors do not provide any evidence for a putative budding step. While BICD2 knockdown accumulates virus in the Golgi, and this may be caused by failure to bud, it would also be possible that the BICD2 and dynein simply prevent dissemination of the virus from a secretory organelle such as the Golgi, thereby keeping it in place to facilitate nuclear entry upon mitosis onset.

Response: We apologize for the confusion. Figure 5C was not meant to demonstrate the failure to bud under BICD2 KD. Our results only show that HPV16 is trapped in the Golgi in the absence of BICD2. We agree that there are multiple interpretations as to what traps the virus in the Golgi, including failure to disseminate from the Golgi. We have now clarified this in the revised manuscript and revised the figure.

3. Potentially, BICD2/dynein may also assist onward trafficking during mitosis, but published work already implicated RanBP10/KPNA2 as adaptors for a dynein mediated transport step at this juncture, and it would be of great interest in addressing how the two adaptors work together or in an alternating fashion. What would cause L2 to switch between adaptors?

Response: We agree that the mechanism of how (or whether) these proteins work together to support infection is of interest. We have now included potential explanations in the discussion section.

4. Also, since the putative interaction site appears to be engaged by several different cellular proteins, I wonder how these interactions would be orchestrated – as I find it very difficult to envision simultaneous interactions at sites that are spatially not at all or not well separated.

Response: We agree that this segment of L2 is densely packed with functional and binding motifs and it is not yet clear how this segment accomplishes this. We now include data demonstrating that the HPV16 L2 peptide can bind both retromer and BICD2 **(new Supplemental Figure 1E**). These results indicate that L2 has the potential to bind both host proteins at the same time, or that different populations of L2 bind to either retromer or BICD2 separately. We now explicitly state in the manuscript that the existence of multiple molecules of L2 in each virus particle makes it likely that each capsid can simultaneously bind BICD2 and retromer, even if each L2 molecule can bind only a single protein at a time.

5. From a virological point of view, it would be of interest to address whether several distinct HPV types would engage the same adaptor(s) for transport. Addressing these (mechanistic) questions experimentally would greatly improve the manuscript would be of interest to a broader readership.

Response: Thank you for this suggestion. We synthesized biotinylated peptides corresponding to the C-terminus of both HPV5 and HPV18. Using the same conditions we used in Figure 2D, we now show that the HPV5 and HPV18 peptides bind purified BICD2 (**new Supplemental Figure 3B and 3C**). As shown in the original manuscript, BICD2 knockdown inhibits infection by these virus types are well. These results suggest that these HPV types also engage BICD2 for transport.

**Minor Issues: Editorial and Data Presentation Modifications**

**Reviewer #1**:

Minor Points:

1. Peptide #4 in Fig 2 is 20 residues, not 19.

Response: Thank you - we have corrected this.

**Reviewer #2**:

Minor Points:

1. The authors used ciliobrevin D (cilio D) to inhibit the ATPase activity of dynein and showed that binding with BICD2 is critical for infection of multiple HPV types. A novel inhibitor Dynarrestin with less toxicity was reported and is worth testing in your system

Response: Thank you for this suggestion. However, due to concerns that these inhibitors disrupt mitosis, as noted above we have removed the ciliobrevin D data from the manuscript.

**Reviewer #3**:

Minor Points:

1. Technically, most of the assays are sound. Given that the proximity ligation assay is the sole line of evidence for transport failures, a second line of investigation such as following the viral DNA by immunofluorescence and colocalization analysis may improve clarity on how severe the phenotypes upon perturbation are.

Response: As suggested, we have now included a second assay to analyze trafficking. Specifically, using PsV containing EdU-labeled DNA, we found that the virus is trapped in the Golgi under BICD2 KD (**Supplemental Figure 4**), in agreement with the PLA data.

2. The referencing looks in part a bit random. While in part numerous publications supporting a statement are referenced, in other places only few and sometimes not the first observations are cited.

Response: Thank you - we have added additional references in a more systematic fashion.
