# Supplementary material for: The BICD2 dynein cargo adaptor binds to the HPV16 L2 capsid protein and promotes HPV infection
Source: PLoS Pathog. 2024 Jun 3;20(6):e1012289. doi: 10.1371/journal.ppat.1012289 (PMC11230635; doi:10.1371/journal.ppat.1012289)
Supplement: Attachment — Submitted filename: Speckhart et al. (Point-by-point response).docx [file ppat.1012289.s006.docx]

We thank the editors for the opportunity to address a few final issues raised by reviewer #3. The other two reviewers are satisfied with our earlier response. The reviewer recognizes that we have shown that BICD2 knockdown interferes with HPV trafficking though the Golgi by reducing dynein binding, but he/she feels that we have not proven our model that this is mediated by binding of BICD2 to L2. Indeed, this model, though not proven, is plausible, most consistent with the available data, provides considerable insight into HPV entry, and will lead to additional experiments and fuller understanding of this complex process. We have revised the discussion to clarify our model and highlight some potential alternative explanations. The specific comments of the reviewer are addressed below.

1. If BICD2 binds to penetrated L2 and is responsible for transport, why are the effects on endosomal retention so small particularly, if it would associate already in early endosomes? This means, that there are at least additional adaptors facilitating routing of the virus. Why didn’t the authors attempt to do a double knockdown of BICD2 and BICDR2, if those are considered to be the main dynein adaptors at this point? Also the new data on EdU labelled viral DNA (Suppl. Fig.2) does not support any early endosome delay.

As the reviewer recognizes, the major effect is on retention in the TGN, which is the main focus of the paper. In fact, we have calculated the fold-change in L1 PLA signal in control compared to BICD2 knockdown cells, and showed that at 24 and 32 hpi, the fold-increase in the knockdown cells in the TGN is two to three times higher than in EEA1 compartment (these numbers are now included in the results). Thus, we are justified in focusing on the later event, namely TGN exit, as it is the most dramatic effect. Regarding the modest endosome accumulation, particularly at earlier times, this could be due to incomplete knockdown of BICD2 or to the existence of other dynein adaptors in the endosome, as we stated in the prior version and emphasized in this revision. Neither possibility detracts from our main message, that BICD2 serves as a dynein adaptor required for passage through the TGN/Golgi. Thus, although interesting, we consider further analysis of the modest endosome block, such as combined knockdown or BICD2 and BICDR2 or additional EdU studies, as not pertinent to our major conclusions.

2. Alternatively or additionally, the effects of BICD2 knockdown may account for general transport defects along the endosomal system or within endosome to Golgi transport. It is unfortunate that the epitopes of the interacting peptide cannot be resolved from residues crucial for penetration and retromer binding, but this would have resolved the question.

Although we have not ruled out a general transport defect, we show that the cell cycle is not substantially perturbed by BICD2 knockdown. Furthermore, postulating a more general defect ignores the finding by co-IP, L2 peptide pulldowns, and PLA that HPV associates with BICD2 at a time point consistent with the blockade to TGN exit. This is now discussed more fully in the revised manuscript. In addition, we are finalizing another manuscript reporting another way of interfering with the HPV-BICD2 association in the TGN in the absence of BICD2 knockdown, namely Rab6a knockdown, which also disrupts the HPV-dynein association and blocks HPV exit from the TGN. These results provide independent confirmation that BICD2 plays a crucial role as a dynein adaptor in mediating TGN exit. Finally, even if the defect is more general, the fact remains that BICD2 knockdown perturbs HPV-dynein interaction and HPV trafficking. These issues and alternative explanations for our results are discussed more fully in the discussion.

3. Relying entirely on PLA stainings was another critical point. The authors now provide images following EdU labelled viral DNA. To my surprise (given the considerable signal for EdU), there is no signal in the nucleus and little overlap with the TGN for the scrambled siRNA, and inly incremental increase in BICD2 depleted cells? This inspires little confidence that their model would be correct. One would have expected a notable drop in intranuclear localisation. Where is the viral DNA at this point?

The new EdU data confirm our conclusion from PLA that BICD2 knockdown increases HPV localization in the TGN. The reviewer is correct that we do not see nuclear staining at 24 h in control cells, perhaps suggesting that infection is relatively slow in our cells. Because we do not detect nuclear signal in the control cells, we are puzzled by the reviewer’s comment that “One would have expected a notable drop in intranuclear localization [in knockdown cells]”, because we cannot assess this. Overall, we do not understand why the EdU results diminishes the reviewer’s confidence in the model.

In addition to the responses above, we have gone through the discussion carefully, and added caveats and alternative explanations where appropriate. We have also consolidated and expanded our discussion of previous work on BICD2 and trafficking of cellular cargos.
